# Osteopontin-4 (OPN-4) Suppresses Tumor Progression Features Whilst Sensitizing c643 Anaplastic Thyroid Cells to Sorafenib

**DOI:** 10.3390/biomedicines14050989

**Published:** 2026-04-25

**Authors:** Gabriela Ribeiro Silva, Amanda Lewis Rubim, Flavia da Cunha Vasconcelos, Luciana Bueno Ferreira, John Greenman, Etel Rodrigues Pereira Gimba

**Affiliations:** 1 Programa de Pós-Graduação em Ciências Biomédicas, Fisiologia e Farmacologia, Instituto Biomédico, Niterói CEP 24210-130, Brazil; 2 Grupo de Hemato-Oncologia Molecular, Coordenação de Pesquisa, Instituto Nacional de Câncer, Rio de Janeiro CEP 20230-130, Brazil; 3 Divisão de Laboratórios Especializados—Coordenação de Assistência COAS, Instituto Nacional de Câncer, Rio de Janeiro CEP 20230-130, Brazil; 4 Departamento de Genética, Instituto de Ciências Biológicas e da Saúde, Universidade Federal Rural do Rio de Janeiro, BR-465, Km 07, Seropédica, Rio de Janeiro CEP 23897-000, Brazil; 5 Centre for Biomedicine, Hull York Medical School, University of Hull, Hull HU6 7RX, UK; 6 Departamento de Ciências da Natureza, Universidade Federal Fluminense, Rua Recife 1–7, Bela Vista, Rio das Ostras CEP 28895-532, Brazil

**Keywords:** osteopontin, splicing isoforms, splicing, OPN-4, spheroids, anaplastic thyroid carcinoma, microfluidic device

## Abstract

**Background/Objectives**: Anaplastic thyroid carcinoma (ATC) is one of the most aggressive and lethal forms of malignant neoplasm of the endocrine system, and osteopontin (OPN) has been shown to be aberrantly expressed in this tumor type. Among the five OPN splicing isoforms (OPN-SI), OPN-4 has been recently reported in several tumor types, including ATC, but its functional role(s) have not yet been elucidated. **Methods**: To characterize OPN-4 roles in ATC cells, OPN-4 was ectopically overexpressed in the c643 ATC cell line, generating the c643/OPN-4 cells. OPN-roles were evaluated by cell functional assays, including cell proliferation and viability, using Carboxyfluorescein Succinimidyl Ester (CFSE), crystal violet, and trypan blue assays. For migration, clonogenicity, cell cycle and apoptosis assays were used. For assessment, c643/OPN-4 cells were cultured in two-dimensional (2D) monolayers or three-dimensional (3D) spheroids with the latter being maintained in a bespoke microfluidic system. **Results**: OPN-4 overexpression led to a significant reduction in cell proliferation, viability, migration and clonogenicity. c643/OPN-4 cells displayed a significant accumulation in the G0/G1 phase and a decrease in the S phase of the cell cycle; however this did not affect cell death or the expression levels of other OPN-SI. In a spheroid model of c643/OPN-4 cells, no significant differences were found in spheroid size or viability when compared to those formed by control cells. Notably, OPN-4 overexpression enhanced the effects of sorafenib on cell viability under dynamic treatment conditions involving continuous perfusion. **Conclusions**: These early findings point to the fact that OPN-4 may reduce some aspects of tumor progression features in ATC cells and open new avenues for investigating OPN-4 as a biomarker of therapeutic response in personalized treatment strategies.

## 1. Introduction

Thyroid cancer (TC) is the most common malignant tumor of the endocrine system, ranking seventh among the most diagnosed cancers worldwide, in both sexes in 2022 [1]. TC exhibits a broad spectrum of clinical behavior, ranging from slow-growing tumors with high remission rates and low mortality, to highly aggressive forms associated with poor survival outcomes [2]. Anaplastic thyroid carcinoma (ATC), although rare (2–3% of all TC cases), is one of the most aggressive and fatal solid tumors, with a median survival rate of approximately 4 months post-diagnosis, with more than half of the patients already possessing metastasis at the time of diagnosis [3,4].

Due to its complexity and aggressiveness, the treatment of patients with ATC is based on a multimodal approach, including surgery, chemotherapy, and, more recently, targeted therapies guided by the tumor’s mutational profile [5]. Among these, tyrosine kinase inhibitors (TKIs), such as sorafenib, have shown some clinical benefit. Sorafenib, a multi-target TKI, inhibits both intracellular kinases, such as the RAF kinases and transmembrane receptors including the VEGFR family, PDGFR-β, RET, and KIT; it is approved for the treatment of radioiodine-refractory differentiated thyroid cancer (DTC) [6]. However, the overall effectiveness of Sorafenib alone remains limited, with low rates of complete response [7], but a recent study has shown promising data when Sorafenib is combined with the anti-malarial agent quinacrine in both in vitro and in vivo assays [8]. Considering the high mortality associated with ATC and the limited success of current treatment options, there is an urgent need to identify novel therapeutic targets and reliable tumor biomarkers to enable earlier diagnosis and more precise, individualized, treatment strategies.

Osteopontin (OPN) is one of several molecules aberrantly expressed in ATC that contribute to tumor progression. This family of molecules affects a wide range of biological processes, such as bone remodeling, immune regulation, tissue repair, regeneration, cell survival, migration and adhesion. Moreover, it plays a crucial role in regulating osteoclast and osteoblast functions, thereby contributing to the maintenance of bone mass and structural integrity. Finally, OPN has been associated with cardiovascular and inflammatory diseases, as well as with cancer development and progression [9,10,11]. OPN is a phosphoglycoprotein that, through alternative splicing, can generate at least 5 isoforms: OPN-a, OPN-b, OPN-c, OPN-4, and OPN-5 [12]. OPN-a is the full-length variant that includes all seven exons, OPN-b is missing exon 5, and OPN-c is missing exon 4. OPN-4 lacks both exons 4 and 5, while OPN-5 features an extra exon due to the retention of a segment of intron 3 from the canonical isoform [13]. OPN-4 and OPN-5 appear to be co-expressed across some tumor types; however, OPN-4 can also show tumor-specific expression patterns. Based on these observations, we hypothesize that, similarly to other OPN splice variants, OPN-4 contributes to key aspects of tumor progression [14].

Although most studies on OPN have focused on papillary thyroid carcinoma, the most common thyroid cancer subtype, emerging evidence indicates that OPN also plays a critical role in the molecular mechanisms of ATC. High expression of various OPN splice variants has been observed in both primary tumor tissues and lymph node metastases of ATC, suggesting an association with metastatic potential and tumor aggressiveness [15].

Given that the functional roles of OPN-4 have not been previously investigated in any cancer model and that all five OPN-SI are expressed at similar levels in ATC cell lines [14], this study aimed to investigate specifically how OPN-4 overexpression affects the biological responses of an ATC cell line.

## 2. Materials and Methods

### 2.1. Plasmid Constructs

The plasmid construct pCR3.1/OPN-4, encoding the OPN-4 protein, was generated by GenScript Biotech Corporation, Piscataway, NJ, USA. The full-length complementary DNA (cDNA) of OPN-4 (Pubmed Accession Number: NM_001251829.2) was cloned in the pCR3 vector, between the BamHI and XhoI multicloning sites. The plasmid pCR3.1/Empty Vector (EV), which does not contain any cloned cDNA, was used as a control.

### 2.2. Cell Culture

The c643 cell line, kindly provided by Dr. Paula Soares from the Institute for Research and Innovation in Health (I3S), Portugal, was used as a cell line model to examine the putative roles of OPN-4 in ATC cells [16]. The cells were maintained in RPMI-1640 Medium (RPMI) (Sigma-Aldrich, St. Louis, MO, USA), supplemented with 10% (*v*/*v*) fetal bovine serum (FBS) (Gibco), Penicillin/Streptomycin (P/S) (100 U/mL—Gibco, Grand Island, NY, USA), and amphotericin B (100 µg/mL—Gibco). Cells were incubated in a humidified atmosphere at 37 °C with 5% CO_2_ *v*/*v*, following the conditions recommended by the American Type Culture Collection (ATCC, Manassas, VA, USA).

Both plasmids, pCR.1/OPN-4 and pCR3.1/EV, were transfected into the c643 cells using the Lipofectamine 2000 transfection reagent protocol following the guidelines provided by the manufacturer (Invitrogen, Carlsbad, CA, USA). Twenty-four hours after transfection, the c643 cells were cultured in the presence of 600 μg/mL of geneticin (G418) for the selection of cells that incorporated the vectors. Cells overexpressing OPN-4 were named c643/OPN-4, while the control cells transfected with an empty vector were named c643/EV.

### 2.3. Reverse Transcription-Quantitative Real Time PCR (RT-qPCR)

Total RNA from c643/OPN-4 and c643/EV cells was extracted using the TRIzol™ reagent, according to the manufacturer’s instructions (Invitrogen). The cDNA synthesis was performed using 1.0 μg of each total RNA sample following the protocol of the ImProm-II™ Reverse Transcription System kit (Promega Corporation, Madison, WI, USA). Gene expression levels were evaluated using the quantitative Polymerase Chain Reaction (qPCR) and SYBR Green detection system (SYBR™ Green PCR Master Mix, Applied Biosystems, Foster City, CA, USA). Glyceraldehyde-3-phosphate dehydrogenase (GAPDH) was used as housekeeping gene. Each PCR reaction mixture (10 μL) consisted of 5 μL SYBR Green, 200 ng of cDNA, and a total of 2.5 μL of each specific oligonucleotide primer pair (forward and reverse), each at a final concentration of 0.8 μM. The cycling conditions included an initial incubation at 50 °C for 2 min, followed by 5 min at 95 °C, then 40 cycles of 30 s at 95 °C, 30 s at 60 °C, and 30 s at 72 °C. This was followed by a melting curve analysis, which ranged from 55 to 90 °C with a heating rate of 0.2 °C/s and continuous fluorescence measurement. The relative expression levels of the transcripts were calculated using the relative quantification method, employing the 2^−ΔΔCT^ method.

### 2.4. Carboxyfluorescein Succinimidyl Ester (CFSE) Assay

To evaluate cell proliferation rates, the CellTrace™ CFSE Cell Proliferation Kit (Thermo Fisher Scientific, Waltham, MA, USA) was used. The cells were counted, resuspended at a final concentration of 1 × 10^6^ cells/mL and then incubated at 37 °C for 20 min with 0.2 μM CFSE protected from light. Afterwards, 5 mL of RPMI medium supplemented with 10% (*v*/*v*) FBS was added and incubated for an additional 5 min at 37 °C to remove excess dye. Cells were then washed with PBS, resuspended in complete RPMI medium, and plated at a density of 4 × 10^4^ cells per well in 6-well plates. As a control, cells were separated at the 0 h time point, indicating maximum fluorescence, and cells without CFSE, showing autofluorescence. At each experimental time point, cells were trypsinized and analyzed at 488 nm using the FL1 detector on a flow cytometer.

### 2.5. Trypan Blue Exclusion Assay

The cell viability of c643/OPN-4 cells was assessed using the trypan blue exclusion assay. As for the CFSE assay, 4 × 10^4^ cells were seeded in a 6-well plate and at each experimental time point, cells were trypsinized, suspended in RPMI medium, and then 10 μL of the suspension was diluted with 10 μL of trypan blue solution (0.4% *w*/*v*) and quantified using a Neubauer chamber.

### 2.6. Colony Forming Assay

A total of 5 × 10^3^ cells were seeded into the wells of 6-well plates and incubated until colony formation was observed approximately after 13 days. At the end of this period, colonies were washed with PBS (1×) and fixed with ethanol for 5 min at room temperature. After washing with PBS (1×), colonies were stained with 0.5% (*w*/*v*) crystal violet for 1 h. Subsequently, colonies were washed with distilled water and air-dried at room temperature. Colonies were photographed and then dissolved in 1 mL of 33% (*v*/*v*) acetic acid, followed by absorbance measurement at 595 nm using a spectrophotometer.

### 2.7. Transwell Migration Assays

To evaluate the migration properties, 4 × 10^4^ cells were seeded in each well of a Transwell^®^ polycarbonate membrane (8 µm pore size; Corning Incorporated, Corning, NY, USA) and cultured in 200 μL of RPMI medium without FBS. In the lower chamber, 600 μL of RPMI medium supplemented with 10% (*v*/*v*) FBS was used as a chemoattractant stimulus. Cells were cultured for 24 h in a humidified incubator at 37 °C with 5% CO_2_. Cells that migrated through the membrane were observed on the underside which were fixed and stained using the panoptic staining kit (Laborclin, Produtos para Laboratórios Ltd.a., Pinhais, PR, Brazil). Four different fields of the insert were photographed, and the number of migrating cells was counted using ImageJ software version 1.53 (NIH, Bethesda, MA, USA).

### 2.8. Scratch Wound Healing Assay

Cell migration properties were also evaluated using the scratch wound healing assay. The cells were seeded at 4 × 10^5^ cells per well in 12-well plates. After 24 h of cell adhesion, a confluent monolayer was formed, and a scratch was created using the tip of a 200 µL pipette. At 0 h time point (immediately after scratching) and at 6 h post-scratch, the cell plates were photographed, and migration was analyzed using ImageJ software with the Wound Healing Size Tool plugin. The 6 h time frame was chosen based on a previous study [17]. The percentage of wound closure was calculated by subtracting the wound area after 6 h (A 6 h) from initial wound area (A 0 h), dividing the result by initial wound area (A 0 h), and then multiplying by 100.

### 2.9. Cell Cycle

To analyze the cell cycle, 4 × 10^4^ cells were seeded into the wells of a 6-well plates. After each experimental time point, the cells were trypsinized and resuspended in propidium iodide (PI) with Triton (50 μg/mL PI, 4 mM citrate buffer, 0.3% (*v*/*v*) Triton X-100) and RNase (100 μg/mL Ribonuclease A Sigma-Aldrich and 40 mM citrate buffer) and then incubated for 15 min at room temperature. The cells were examined using a flow cytometer with detection at 585 nm; data were analyzed using FlowJo 10.10.0 cytometry analysis software (BD Biosciences, San Jose, CA, USA).

### 2.10. Evaluation of Cell Death by Annexin V/Propidium Iodide (PI) Assay

To assess cell death, a density of 4 × 10^4^ cells/well in 6-well plates was seeded. At each experimental time point, the supernatant containing the dead cells was collected, and the adherent cells were removed by enzymatic dissociation with trypsin. The cell pellet was resuspended in binding buffer (10 mM HEPES, 140 mM NaCl, 2.5 mM CaCl_2_ pH 7.4) and annexin V according to the manufacturer’s introduction (Sigma-Aldrich, St. Louis, MO, USA) and then incubated for 15 min at room temperature. After that, PI (50 μg/mL) was added. The fluorescence of Annexin V conjugated to Alexa488 and PI were detected, respectively, through the FL1 channel at 530/540 nm, and the FL3 channel at 613/620 nm by flow cytometry. Data analysis was performed using FlowJo 10.10.0 cytometry software (BD Biosciences, San Jose, CA, USA). Cell death rates were determined by calculating the sum of the percentage of cells that were positive for Annexin V and negative for propidium iodide (Annexin V^+^/PI^−^), which corresponds to early apoptotic cells, and those that were positive for both Annexin V and PI (Annexin V^+^/PI^+^), indicative of late apoptosis or secondary necrosis.

### 2.11. 3D Cell Spheroid Generation

A total of 3 × 10^4^ c643/OPN-4 and c643/EV cells were seeded with complete medium into 96-well ultra-low attachment (ULA, Corning Incorporated, Corning, NY, USA) plates to promote cell spheroid formation. Spheroid growth was monitored over 13 days, with imaging conducted on days 1, 3, 5, 7, 10, and 13. To support maturation, spheroids were allowed to grow for three days before removing 100 μL of media and replacing with fresh, this was subsequently performed every three days.

### 2.12. On-Chip Setup

Spheroids were cultured until day 10, at which point they were transferred to a previously described microfluidic chip device for further experimentation [18]. The chip was fabricated from poly(methyl-methacrylate) (PMMA) (Kingston Plastics, Hull, UK), having designed the components using Autodesk AutoCAD 2025 software (Autodesk Inc., San Rafael, CA, USA), and manufactured using a laser cutter (LS6840 Pro, HPC Laser, Halifax, UK) [19].

Prior to cell loading, chips were sterilized with 70% (*v*/*v*) ethanol for 5 min and then primed with sterile culture medium. Spheroids were collected from ULA plates and introduced into the chip under sterile conditions in a class II biological safety cabinet, with three spheroids placed in each device. A 20 mL syringe containing complete culture medium was connected to the chip inlet via ethylene tetrafluoroethylene (ETFE) tubing, and perfusion was maintained at a constant flow rate of 2 μL/min.

The microfluidic device was kept at 37 °C in an “egg incubator” and exposed to dynamic treatment with 6, 25 and 100 µM of sorafenib for 48 h. Following treatment, spheroids were carefully retrieved from the chip and transferred to 96-well plates for downstream analyses, including the CellTiter-Glo^®^ (CTG) assay and FDA/PI staining.

### 2.13. Measuring Cell Spheroids

To assess spheroid size, images were captured using the GelCount™ system (Oxford Optronix Ltd., Adderbury, UK) on days 1, 3, 5, 7, 9, 10, and 13. Fiji (ImageJ) 2.x, build 2025 software was used to measure the vertical, horizontal, and diagonal diameters of each spheroid, enabling calculation of an average diameter over time. Measurements were calibrated using the known diameter of the well, and spheroid sizes were recorded in millimeters.

### 2.14. Spheroid Morphological Analysis

The spheroid morphology was evaluated in terms of size and circularity. Images were captured with the GelCount™ system as described above. Circularity is a geometric parameter that reflects how closely a shape resembles a perfect circle. A circularity value of 1.0 corresponds to an ideal circle, while values approaching 0.0 indicate increasingly irregular or elongated shapes. In the context of spheroids, circularity is commonly used to evaluate surface uniformity, as spheroids with poor circularity often exhibit irregularities along their perimeter. Circularity index was analyzed using the FIJI/ImageJ software.

### 2.15. CellTiter-Glo^®^ (CTG) 3D Cell Viability Assay

A total of 100 μL of culture medium containing the spheroids, previously incubated for the designated experimental time, was transferred to an opaque white-walled 96-well plate (Corning). The CTG reagent was thawed at room temperature, and 100 μL was added to each well. The plate was placed on a plate shaker at 1200 rpm for 5 min to promote cell lysis, followed by a 25 min incubation at room temperature. Luminescence generated by the reaction was measured and recorded using a luminometer (FLUOstar Omega (BMG LABTECH GmbH, Ortenberg, Germany).

### 2.16. FDA/PI Staining Protocol of Spheroids

FDA/PI Staining was used to assess cell viability and death in spheroids. Three spheroids were transferred from ULA plates to a μ-Slide 8 Well plate (Corning), and excess medium was carefully aspirated. The spheroids were washed with 200 μL of ice-cold PBS (1×), and all liquid was removed prior to staining. A staining solution (16 μL of 5 mg/mL FDA and 2 μL of 100 mg/mL PI in 10 mL PBS) was prepared, and 200 μL was added to each well. After a 15 min incubation at 37 °C in the dark, the solution was removed, and wells were washed three times with PBS (1×). The plate was wrapped in aluminum foil and transported to the microscope suite. Spheroids were imaged immediately using a fluorescence microscope (Carl Zeiss AG, Oberkochen, Germany) with filters for FDA (495 nm/517 nm) and PI (596 nm/614 nm). FDA stains the cytoplasm of viable cells green (live), while PI stains the nuclei of non-viable (dead) cells red.

### 2.17. Sorafenib Spheroid Treatment

Once c643/EV and c643/OPN-4 spheroids reached 10 days of maturation, the culture medium was removed from the wells and sorafenib (Sino Biological, Beijing, China) was added. Sorafenib was dissolved in dimethyl sulfoxide (DMSO), then diluted in media to give final concentrations of 100, 25 and 6 μM. A volume of 100 μL/well of each sorafenib concentration, as well as control conditions (medium only or DMSO-containing medium at 0.5% *v*/*v*, corresponding to the concentration of DMSO in the 100 μM sorafenib solution), was added to the appropriate wells in ULA plates, called static treatment. For the on-chip treatment, called dynamic treatment, undergoing constant culture media perfusion, the same concentrations of solutions were loaded into syringes and introduced into the microfluidic chip. ULA plates were incubated at 37 °C with 5% CO_2_, and the chip was maintained at 37 °C on egg incubator. Both treatment modalities were applied for 48 h.

### 2.18. Statistical Analysis

All data were presented as mean or median ± standard deviation (SD). Differences between groups were evaluated using the student *t*-test, the Wilcoxon test, the Kruskal–Wallis test, or the ANOVA test, using GraphPad Prism v.8.0.2^®^ software as appropriate. Values of *p* ≤ 0.05 were considered statistically significant. * *p* ≤ 0.05; ** *p* ≤ 0.01; *** *p* ≤ 0.001.

## 3. Results

### 3.1. OPN-4 Overexpression Decreases Proliferation and Viability Rates in c643 Cell Line

**Figure 1:**
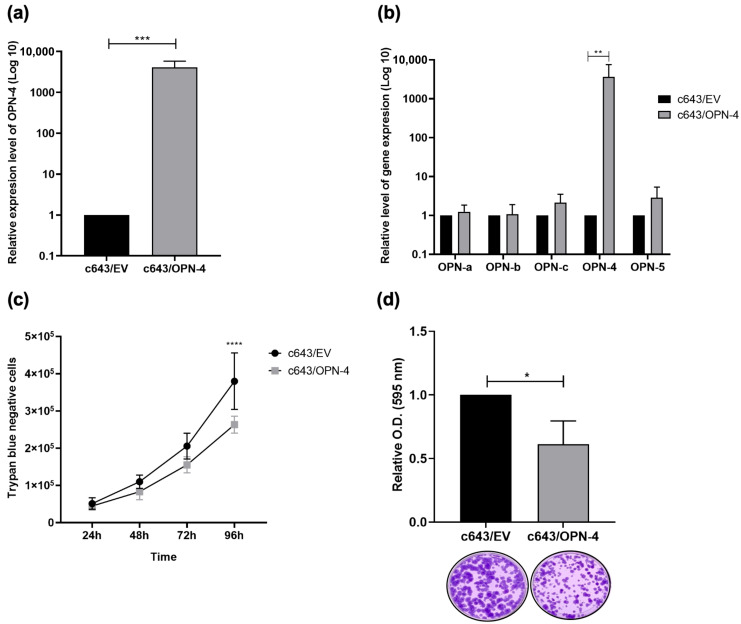
OPN-4 overexpression and effects on viability. The representative anaplastic thyroid carcinoma cell line c643 was stably transfected with the pCR3.1/EV or pCR3.1/OPN-4 plasmid. (**a**) Quantitative reverse transcription polymerase chain reaction (RT-qPCR) was used to determine the mRNA expression levels of OPN-4 and (**b**) OPN-SI in the c643/OPN-4 and c643/EV cell lines. Relative expression levels were calculated using the 2^−ΔΔCq^ method, with GAPDH used as the housekeeping gene. Statistical analysis was performed using the Wilcoxon and Kruskal–Wallis test from 7 and 6 independent experiments, respectively. (**c**) Cell viability was assessed using the trypan blue exclusion assay, conducted across 6 independent experiments. (**d**) Clonogenicity was analyzed after 13 days in culture by staining with crystal violet. Optical density (OD) was measured at 595 nm in 4 independent experiments. Error bars represent the mean ± SD. Statistical analysis was performed using ANOVA and Wilcoxon tests, respectively. * *p* < 0.05, ** *p* < 0.01, *** *p* ≤ 0.001, **** *p* < 0.0001.

The OPN-4 cDNA was cloned into the pCR3.1 plasmid expression vector by GenScript Biotech and the ectopic overexpression of OPN-4 was validated by RT-qPCR The c643/OPN-4 cells exhibited an approximately 4000-fold increase in OPN-4 transcript levels compared to the c643/EV control cells (*p* = 0.0006) (Figure 1a).

To investigate whether the expression of other OPN-SI was modulated in c643/OPN-4 cells, the relative expression levels of OPN-a, OPN-b, OPN-c, and OPN-5 were similarly analyzed by RT-qPCR using specific primers. No change was observed in the relative expression levels of these isoforms in response to OPN-4 overexpression (Figure 1b).

To assess cell viability in c643/OPN-4 cells, trypan blue exclusion and colony formation assays were performed. The trypan blue exclusion assay revealed a 30.71% reduction in the number of c643/OPN-4 cells at the 96 h time point when compared to c643/EV control cells (*p* < 0.0001) (Figure 1c). It was also found that the c643/OPN-4 cells exhibited a 38.7% reduction in clonogenicity in relation to control cells (*p* = 0.0286) after 13 days of cell culture (Figure 1d).

**Figure 2:**
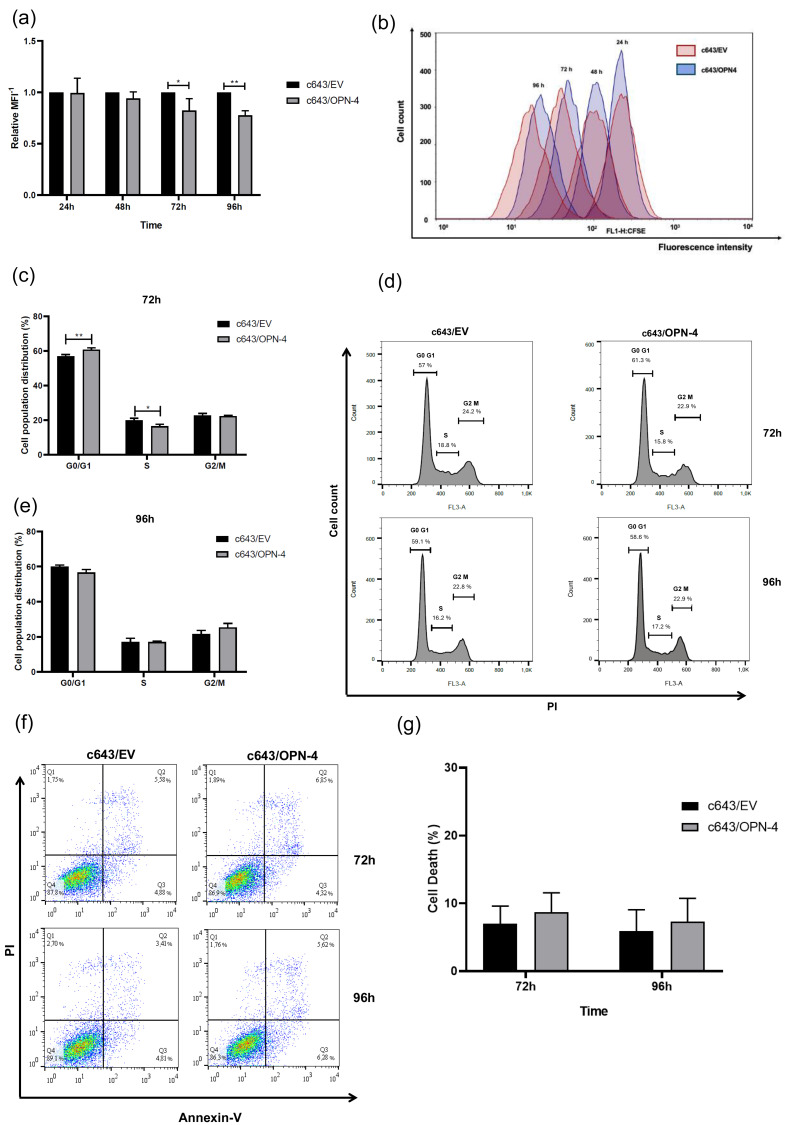
Proliferation and cell cycle distribution profile of cells overexpressing OPN-4. (**a**) The kinetic analysis of proliferation in c643/OPN-4 and c643/EV cells was assessed using the mean fluorescence intensity (MFI) of CFSE, with values represented as 1/MFI. (**b**) Representative histogram from one of four independent experiments is shown. The *x*-axis indicates the fluorescence intensity detected in the FL1 channel at 488 nm, while the *y*-axis represents the number of cells. Results were analyzed using FlowJo™ v10.8 (BD Life Sciences). c643/OPN-4 and c643/EV cells were stained with propidium iodide (PI), and fluorescence intensity was measured in the FL3 channel at 585 nm at the 72 (**c**,**d**) and 96 h (**d**,**e**) The histogram on the right shows data from a representative experiment out of three independent trials. The *x*-axis represents the fluorescence intensity emitted by PI, while the *y*-axis represents the number of cells. Statistical analysis was performed using one-way ANOVA. * *p* < 0.05 and ** *p* < 0.01. (**f**) Cell death percentage was determined using flow cytometry with Annexin V and propidium iodide (PI) staining. Cells positive for Annexin V but negative for PI (Annexin V^+^/PI^−^) and cells positive for both Annexin V and PI (Annexin V^+^/PI^+^) were considered dead. Colors indicate cell density in the plot: blue color represents low cell density, green indicates intermediate cell density, and yellow/red show high cell density, highlighting regions with more cells. (**g**) The histogram is a representative assay from a total of four independent experiments. Error bars represent the mean ± SD. Statistical analysis was performed using one-way ANOVA.

A CFSE assay was performed to assess c643/OPN-4 cell proliferative potential. At the 72 h and 96 h time points, lower proliferation rates were observed for the c643/OPN-4 cells having a 17.8% (*p* = 0.0225) and 22.5% (*p* = 0.0058) decrease, respectively, in the proliferation index compared to the c643/EV control cells (Figure 2a,b).

Since changes in cell proliferation rates in response to OPN-4 overexpression were observed at 72 h and 96 h, cell cycle distribution was analyzed by flow cytometry at these same time points. At 72 h, the OPN-4 overexpressing cells exhibited a 3.9% increase in the percentage of cells in G0/G1 (*p* = 0.0098), and a 3.4% decrease in the S phase (*p* = 0.0278), with no significant changes observed in the proportion of cells in the G2/M phase compared to c643/EV cells (Figure 2c,d). Thus, OPN-4 overexpression induced cell cycle arrest, promoting exit from S phase and G0/G1 accumulation. However, no changes were observed at 96 h in the cell cycle distribution between c643/OPN-4 and c643/EV cells (Figure 2d,e).

To validate these findings and determine whether OPN-4 overexpression is associated with an antiproliferative rather than a pro-death effect, cell death rates at the same experimental time points were assessed. Although a reduction in proliferation and viability rates was observed in c643/OPN-4 cells, no significant differences in cell death rates were detected between c643/OPN-4 and c643/EV cells (Figure 2f,g), indicating that the observed effects are most likely due to decreased proliferation rather than increased cell death.

### 3.2. OPN-4 Overexpression Suppresses Migration Properties in c643 Cell

**Figure 3:**
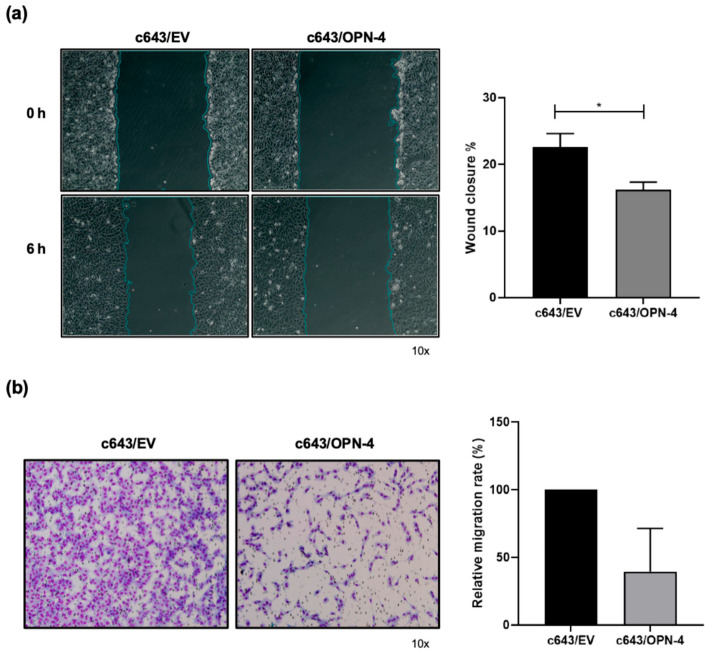
Analysis of migration in cells overexpressing OPN-4. (**a**) In the images on the left, cells immediately after wounding (0 h) and 6 h later were captured. Bar graphs on the right correspond to the quantification of % wound closure. The wound closure area was quantified using ImageJ software (bar graphs on the right). The percentage of wound closure was calculated using the formula (A 0 h–A 6 h/A 0 h) × 100. The time A = 0 h represents the initial wound area and A = 6 h represents the wound area after 6 h. The blue line represents the boundary of the wound area (scratch) identified by the image analysis software. It delineates the cell-free region at each time point (0 h and 6 h), allowing accurate quantification of wound area reduction over time. Statistical analysis was performed using Student’s *t*-test. The graph represents the results of a scratch wound healing assay from one representative experiment out of three independent experiments. (**b**) Cell migration was assessed using Transwell^®^ inserts with an 8 μm pore size. Images of four fields from the inserts were captured using an Axio Observer.Z1 microscope equipped with an Axio CamHRc and Axio Vision Release 8.2 Image Analyzer (Carl Zeiss, Oberkochen, Germany). In the images on the left, migrated cells were stained using a rapid panoptic kit, and the results are representative out of three independent experiments. The number of cells that migrated through the membrane was quantified using ImageJ software. Statistical analysis was performed using the Wilcoxon test. Error bars represent the mean ± SD. Statistical analysis was performed using the Wilcoxon test. * *p* < 0.05.

To investigate the impact of OPN-4 overexpression on cell movement, migration assays were conducted. In Figure 3a, it was found that c643/OPN-4 cells displayed a significant reduction in migration rates in relation to control cells. At the 6 h time point, c643/EV cells closed 22.5% of the wound, while c643/OPN-4 cells only closed 16.1%.

In transwell migration assays, the c643/OPN-4 cells similarly migrated around 60% less when compared to the c643/EV cells, although this difference did not reach statistical significance (*p* = 0.25) (Figure 3b).

### 3.3. OPN-4 Overexpression Alters Early Spheroid Formation and Circularity

To determine whether the effects observed on cell viability rates in 2D culture were recapitulated in 3D culture, cell spheroids were generated from c643/OPN-4 and c643/EV cells, and spheroid viability was assessed over time using two complementary assays (Figure S1a–h). No significant differences were observed in metabolic activity (Figure S1a) or live/dead cell proportions (Figure S1b) between the OPN-4 overexpressing cells and the empty vector control cells at any time points analyzed.

**Figure 4:**
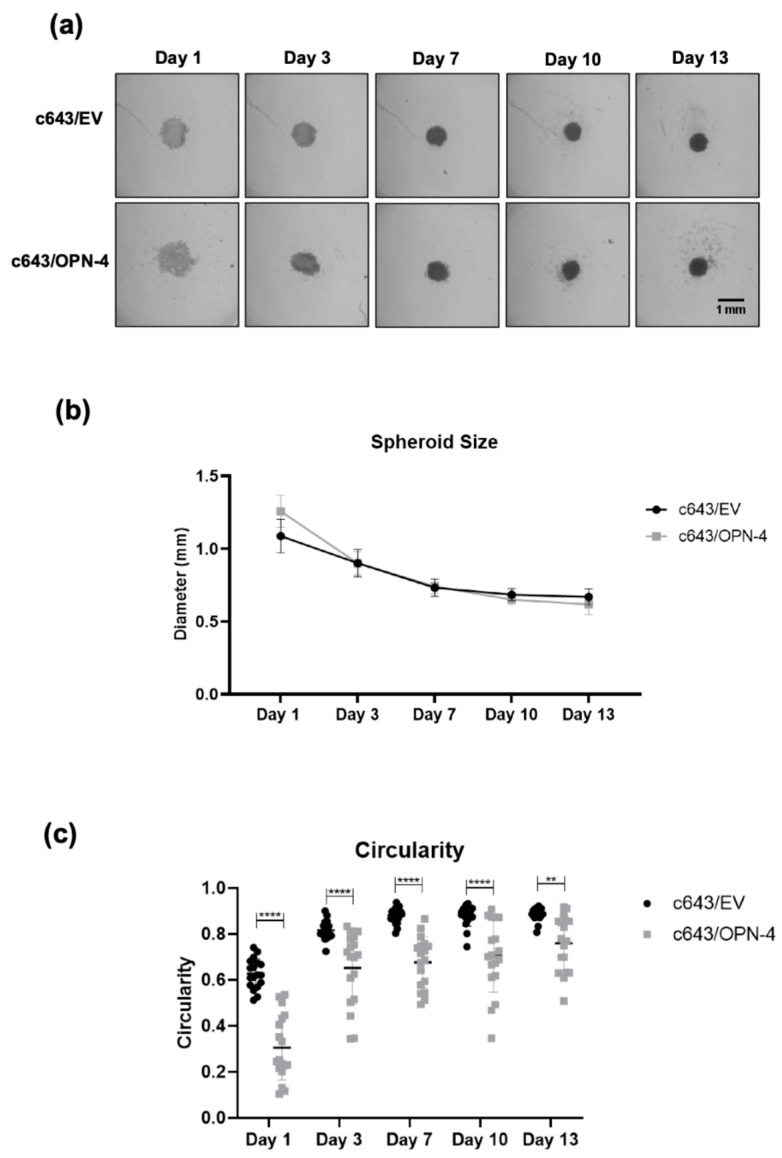
Formation and morphological analysis of 3D tumor spheroids from OPN-4-overexpressing cells. (**a**) Representative brightfield images of c643/EV and c643/OPN-4 spheroids captured on 1, 3, 7, 10, and 13 days. Spheroids were generated in ULA plates and imaged using a light microscope. Images represent one of three biological replicates. (**b**) Spheroid diameter (in millimeters), quantified using Fiji/ImageJ. Data represents the mean ± SD from three independent experiments. Statistical analysis was performed using two-way ANOVA followed by Tukey’s multiple comparisons test. (**c**) Quantification of spheroid circularity using Fiji/ImageJ, comparing c643/EV and c643/OPN-4 groups. Data represents 18 spheroids per condition. Statistical analysis was performed using two-way ANOVA with Bonferroni’s multiple comparisons test. ** *p* < 0.01, **** *p* < 0.0001. Scale bar = 1 mm.

Spheroid size was also monitored over time, and a gradual reduction in average diameter was observed in both c643/OPN-4 and c643/EV cell spheroids (Figure 4a,b). Initially, c643/OPN-4 spheroids had an average diameter of 1.26 mm, while c643/EV spheroids measured 1.09 mm. Reduction was observed until day 7, after which spheroid sizes stabilized and remained constant until day 13. At the end of the experiment, the average diameters were 0.62 mm and 0.67 mm for the c643/OPN-4 and c643/EV spheroids, respectively (Figure 4b).

To assess spheroid formation, particularly in terms of its surface uniformity, spheroid circularity analysis was conducted. The c643/OPN-4 spheroids exhibited significantly lower circularity than c643/EV spheroids throughout the entire observation period (Figure 4c). This difference was statistically significant from day 1 (*p* < 0.0001) and remained evident throughout day 13 (*p* = 0.0014).

### 3.4. OPN-4 Overexpression Enhances the Response to Sorafenib in Spheroid-on-Chip Treatment

Clinical studies have shown that sorafenib is effective in treating patients with DTC, particularly those unresponsive to radioactive iodine [20,21]. Although ATC is an undifferentiated malignancy, patients also exhibit refractoriness to radioactive iodine therapy, suggesting that OPN-4 could modulate sensitivity to Sorafenib treatment. Furthermore, there have been several studies showing that Sorafenib can work in combination with other agents, especially when studied in vitro [8]. Here we used Sorafenib in a preliminary study to show how the 3D model could be used to investigate effects on c643 cell lines ectopically overexpressing OPN-4.

Spheroids were treated with sorafenib at 6, 25, and 100 µM, as the maximum concentration (C-max) for this drug in plasma is reported between 4 and 45 µM [22]. Under static culture conditions, spheroids overexpressing OPN-4 did not show any significant changes in the proportion of live/dead cells at any of the sorafenib concentrations tested, when compared to control spheroids (Figure S2a–f). The DMSO control showed no significant effect on cell viability, even at the highest concentration used (Figure S2c).

**Figure 5:**
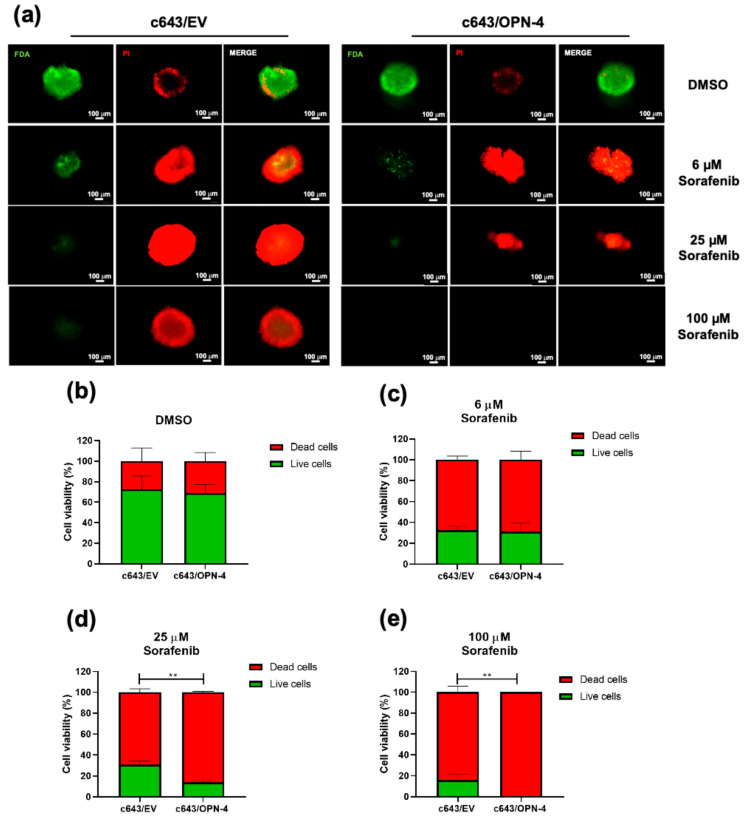
Evaluation of dynamic sorafenib treatment in a microfluidic chip model using OPN-4-overexpressing tumor spheroids. (**a**) Representative fluorescent images of spheroids stained with Fluorescein Diacetate (FDA) and Propidium Iodide (PI) after 48 h of sorafenib treatment. Live cells fluoresce green (FDA), and dead cells fluoresce red (PI). Images were acquired using a fluorescence microscope at 10× magnification. Each image represents the result of a representative assay from a total of three independent experiments performed in triplicate. (**b**–**e**) Quantification of live/dead cell ratios in spheroids treated with DMSO (vehicle control) (**b**), (**c**) 6 µM, (**d**) 25 µM, and (**e**) 100 µM sorafenib, respectively. ImageJ was used to calculate the percentage of viable versus non-viable cells. Statistical analysis was performed using an unpaired *t*-test of three independent experiments. ** *p* < 0.01. Scale bar = 100 µm.

In contrast, spheroids overexpressing OPN-4 when cultured in dynamic treatment conditions showed an enhanced response to sorafenib at both 25 µM and 100 µM, when compared to the c643/EV control spheroids (Figure 5a–e). At 25 µM sorafenib a significant reduction in the percentage of viable cells was observed in the c643/OPN-4 spheroids relative to c643/EV controls (*p* = 0.0011) (Figure 5d). This trend became even more pronounced at 100 µM (*p* = 0.0087) (Figure 5e), where no spheroids were recovered from the c643/OPN-4 cells within the microfluidic chip, suggesting complete spheroid disintegration and/or cell death.

## 4. Discussion

This study aimed to investigate the functional role of OPN-4 in tumor progression-related features and the response to sorafenib, employing the c643 ATC cell line as an experimental platform, although the OPN-4-related effects observed here may be limited to this genomic context instead of being a universal feature of ATC. This is the first report describing this OPN splicing isoform in a cancer context. The overexpression of this isoform suppressed cell proliferation, viability, and migration, evidencing a repressive effect on tumor progression features in this ATC cell line model. Moreover, OPN-4 overexpression sensitized the ATC spheroids to sorafenib under dynamic culture conditions.

OPN is a multifunctional protein widely studied as a prognostic and predictive biomarker due to the correlation of its expression with more invasive tumors, its ability to contribute to tumor progression, and its role in treatment responses [23]. OPN-4 was first described by Lin et al. in 2015 in esophageal carcinoma cells, where overexpression was observed in primary tumor samples compared to matched non-tumoral controls [24].

The expression of OPN isoforms varies widely depending on the type of tumor investigated [9,12]. Although some studies have outlined the expression profile of the OPN-4 splicing isoform, no report has yet explored its potential role in tumor progression in ATC or in any thyroid cancer cell line model.

Overexpressing OPN-4 showed no changes in the expression levels of other OPN-SI, indicating that all the observed cellular effects were most likely due to the OPN-4 overexpression and not to modulation of other OPN splice variants. This contrasts with what is seen in the MBNL family in Myotonic Dystrophy Type 1, where a compensatory mechanism is observed, i.e., the loss of MBNL1 function is offset by increased MBNL2 levels [25].

The c643/OPN-4 cells showed a significant decrease in proliferation compared to the c643/EV cells beginning at 72 h, with this effect becoming more pronounced at 96 h when assayed using a CFSE assay. Supporting this result was the fact that a lower number of OPN-4 overexpressing cells was observed at 96 h and with a reduced clonogenic capacity by the end of day 13 of culture, when compared to the parent c643 line.

We also observed that at 72 h time point the c643/OPN-4 cells exited the S phase and accumulated in the G0/G1 phase compared to control cells, indicating a cell cycle arrest. Importantly, no changes in cell death were detected at the same time points. In line with previous observations reporting that Xbp1 expression under IL-3 signaling induces cell cycle arrest without concomitant apoptosis [26], our data indicate that OPN-4 decreases cell proliferation by promoting cell cycle arrest rather than exerting cytotoxic effects.

These findings contrast sharply with previous reports on other OPN-SI. For instance, overexpression of OPN-a in ATC cell lines (including c643 and 8505c) promotes proliferation as early as 48 h, as measured by BrdU incorporation [17]. Similarly, in human endometrial adenocarcinoma cells, OPN-b and OPN-c were both shown to enhance cell proliferation, with OPN-b being the most potent [27]. The inhibitory effect of OPN-4 on proliferation, as demonstrated here, positions it as a functionally distinct isoform that may counterbalance the pro-tumorigenic actions of other OPN variants. OPN-4 could potentially act as a negative regulator of tumor growth in part through cell cycle arrest. The divergent effects reported for the different OPN splice variants suggests that it is the specific cellular context and isoform signaling which influence cell survival, proliferation, migration, and invasion.

Interestingly, OPN-4 overexpression also suppressed cell migration. In the same cell line used in this study (c643), Ferreira and colleagues demonstrated that OPN-a overexpression enhanced MMP-2 activity, indicating increased extracellular matrix (ECM) degradation capacity. Furthermore, OPN-a also has been shown to enhance both motility and migration [17]. OPN-4 lacks both exon 4 and 5, and notably exon 4 is critical for ECM interaction and MMP activation. Exon 4 contains glutamine residues essential for transglutaminase-mediated crosslinking, a process that increases OPN’s affinity for collagen and promotes its polymerization and binding to ECM components [28]. The absence of this exon in OPN-4 likely compromises its ability to polymerize and interact with the ECM, thereby impairing the ECM degradation required for effective migration. In addition, the absence of exons 4 and 5 in OPN-4 alters post-translational modifications as key phosphorylation sites are deleted, which may further impact the structural conformation, stability, and intracellular interactions [12]. As such, OPN-4 probably influences intracellular pathways involved in cytoskeletal organization and cell adhesion, mechanisms sufficient to negatively modulate migration, although the molecular mechanisms underlying these cellular behaviors have not been explored in this study.

Similarly to our findings, it has been previously reported that OPN expression correlated with a favorable prognosis, aligning with roles on inhibiting tumor progression features. For instance, two studies reported that higher OPN levels were linked to better clinical outcomes [29,30]. In medullary thyroid carcinoma (MTC), OPN expression was strongly associated with smaller, typically non-invasive tumors. Additionally, OPN-a was significantly upregulated in these tumors compared to non-tumor tissues, and its overexpression in a MTC derived cell line resulted in reduced cell growth and viability [30]. Similarly, in pancreatic adenocarcinoma, OPN expression correlated with a significant survival advantage, independent of other clinicopathological variables [29]. Taken together with the present data, it is clear that the role of specific molecules in cancer is highly context-dependent, and that their functions can vary substantially according to the biological microenvironment.

To further investigate these effects, particularly in light of the complexity of the tumor, we employed a 3D model. Tumor spheroids more closely replicate the characteristics of in vivo tumor tissue, exhibiting gene expression patterns and phenotypes that are more representative of actual tumors compared to traditional 2D monolayer cell cultures [31,32,33]. The use of a perfusion-based microfluidic device to maintain the spheroids further increases the in vivo-like nature of the system as there is physiological shear stress, a constant supply of nutrients and oxygen, and removal of waste products, distinctly different from the batch culture of 2D cells [18,19,34,35].

Analysis of spheroid circularity revealed that spheroids formed by OPN-4-overexpressing cells consistently exhibited significantly lower circularity compared to controls throughout the 13-day observation period, with differences evident as early as day 1. Lower circularity values are associated with irregularities along the spheroid perimeter, suggesting that OPN-4 overexpression may impair the formation of well-organized structures, and disrupts normal spheroid morphogenesis [36]. These findings support OPN-4 roles in modulating spheroid architecture, potentially contributing to changes in tumor tissue organization and mechanical properties.

Another emerging strategy involves identifying patient subgroups with increased sensitivity to treatment, a concept applicable to almost all malignancies. In the present study, spheroids overexpressing OPN-4 enhanced sensitivity to sorafenib starting at the concentration of 25 µM, but this effect was observed exclusively under dynamic treatment conditions when maintaining the spheroids on a microfluidic device. Plasma concentrations up to 45 µM have been reported in vivo especially when dosing is given in a pulsed manner, thus effects observed in this in vitro system are likely to mimic pharmacological effects [22]. These findings raise the possibility that OPN-4 overexpression may be affected by shear stress that influences cellular sensitivity to sorafenib. Further studies will be needed to determine whether this isoform has predictive value in more complex preclinical or clinical settings, and testing whether this splice variant also modulates the response to other chemotherapeutic drugs or targeted therapies. The enhanced response under flow conditions highlights the relevance of using advanced, dynamic in vitro models, such as microfluidic systems and organ-on-a-chip systems, for more accurate preclinical evaluation of anticancer therapies [34,35,37,38]. Similarly, other biomarkers, including evaluation of BRAF mutational status and AXL expression, have been suggested for risk stratification and for identifying patients with differentiated thyroid cancer who may benefit from more intensive radioactive iodine therapy or alternative therapeutic approaches, such as immunotherapy [39].

In line with previous findings, sorafenib has demonstrated antitumor activity in ATC cell lines, including c643, primarily through dose-dependent reductions in cell viability, induction of apoptosis, and cell cycle arrest [40,41]. These results add to our knowledge by showing that OPN-4 overexpression enhances sorafenib sensitivity under dynamic flow conditions, suggesting that specific molecular contexts may modulate drug response. Nevertheless, the mechanisms responsible for the observed G0/G1 arrest and sorafenib responsiveness are still uncertain, since downstream pathway investigations (such as PI3K/AKT and MAPK via Western blot) should be further investigated.

The observation that OPN-4 overexpression alone, without co-treatment, can sensitize c643 ATC spheroids to sorafenib under microfluidic conditions highlights a potential avenue for refining therapeutic strategies. While these findings are preliminary, they suggest that OPN-4 may act as a modulator of therapeutic response, warranting further investigation in more complex models.

## 5. Conclusions

In summary, these early findings suggest that OPN-4 suppresses tumor progression features in the ATC c643 cell line, in contrast to the well-established oncogenic activity of the canonical isoform OPN-a. Interestingly, OPN-4 overexpression enhanced sensitivity of ATC c643 cell line spheroids to sorafenib under dynamic treatment conditions, which better mimic the physiological environment compared with 2D monolayers. These findings raise the possibility that OPN-4 expression could influence the therapeutic response in ATC cells. Taken together, these early findings point to a possible role of alternative splicing in modulating tumor behavior and highlight the need for further studies to clarify OPN-4’s biological and clinical significance in other cancer models.

## Data Availability

The datasets are archived at https://data.mendeley.com/datasets/tw3rx4wcnn/1 (accessed on 19 April 2026).

## Supplementary Materials

The following supporting information can be downloaded at: https://www.mdpi.com/article/10.3390/biomedicines14050989/s1, Figure S1: Viability analysis of 3D tumor spheroids derived from OPN-4-overexpressing cells.; Figure S2: Evaluation of static sorafenib treatment in plate-cultured OPN-4-overexpressing tumor spheroids.

## Abbreviations

The following abbreviations are used in this manuscript:

OPN	Osteopontin
OPN-4	Osteopontin-4
OPN-SI	OPN splicing isoforms
ATC	Anaplastic thyroid carcinoma
CFSE	Carboxyfluorescein Succinimidyl Ester
TC	Thyroid cancer
TKIs	tyrosine kinase inhibitors
DTC	Differentiated thyroid cancer
FBS	Fetal bovine serum
GAPDH	Glyceraldehyde-3-phosphate dehydrogenase
qPCR	Quantitative polymerase chain reaction
RT-qPCR	Reverse transcription quantitative polymerase chain reaction
PMMA	Poly(methyl-methacrylate)
ETFE	Ethylene tetrafluoroethylene
CTG	CellTiter-Glo^®^
DMSO	Dimethyl sulfoxide
SD	Standard deviation
MFI	Mean fluorescence intensity
PI	Propidium iodide
BrdU	5-bromo-2′-deoxyuridine
ECM	Extracellular matrix
MTC	Medullary thyroid carcinoma

## References

[1] Lyu Z., Zhang Y., Sheng C., Huang Y., Zhang Q., Chen K. (2024). Global Burden of Thyroid Cancer in 2022: Incidence and Mortality Estimates from GLOBOCAN. Chin. Med. J..

[2] Durante C., Costante G., Lucisano G., Bruno R., Meringolo D., Paciaroni A., Puxeddu E., Torlontano M., Tumino S., Attard M. (2015). The Natural History of Benign Thyroid Nodules. JAMA.

[3] Simões-Pereira J., Capitão R., Limbert E., Leite V. (2019). Anaplastic Thyroid Cancer: Clinical Picture of the Last Two Decades at a Single Oncology Referral Centre and Novel Therapeutic Options. Cancers.

[4] Lin B., Ma H., Ma M., Zhang Z., Sun Z., Hsieh I.-Y., Okenwa O., Guan H., Li J., Lv W. (2019). The Incidence and Survival Analysis for Anaplastic Thyroid Cancer: A SEER Database Analysis. Am. J. Transl. Res..

[5] Bible K.C., Kebebew E., Brierley J., Brito J.P., Cabanillas M.E., Clark T.J., Di Cristofano A., Foote R., Giordano T., Kasperbauer J. (2021). 2021 American Thyroid Association Guidelines for Management of Patients with Anaplastic Thyroid Cancer: American Thyroid Association Anaplastic Thyroid Cancer Guidelines Task Force. Thyroid.

[6] Cheng L., Fu H., Jin Y., Sa R., Chen L. (2020). Clinicopathological Features Predict Outcomes in Patients with Radioiodine-Refractory Differentiated Thyroid Cancer Treated with Sorafenib: A Real-World Study. Oncologist.

[7] Recent Advances in the Management of Anaplastic Thyroid Cancer|Thyroid Research|Springer Nature Link. https://link.springer.com/article/10.1186/s13044-020-00091-w.

[8] Abdulghani J., Gokare P., Gallant J.-N., Dicker D., Whitcomb T., Cooper T., Liao J., Derr J., Liu J., Goldenberg D. (2016). Sorafenib and Quinacrine Target Anti-Apoptotic Protein MCL1: A Poor Prognostic Marker in Anaplastic Thyroid Cancer (ATC). Clin. Cancer Res..

[9] Xu W., Bi Z., Lu L., Feng F., Chen L., Zhang C. (2025). Role of Osteopontin in Cancer: From Pathogenesis to Therapeutics (Review). Oncol. Rep..

[10] Viana B.P.P.B., Gomes A.V.P., Gimba E.R.P., Ferreira L.B. (2021). Osteopontin Expression in Thyroid Cancer: Deciphering EMT-Related Molecular Mechanisms. Biomedicines.

[11] Kumari A., Kashyap D., Garg V.K. (2024). Osteopontin in Cancer. Adv. Clin. Chem..

[12] Bastos A.C.S.d.F., Gomes A.V.P., Silva G.R., Emerenciano M., Ferreira L.B., Gimba E.R.P. (2023). The Intracellular and Secreted Sides of Osteopontin and Their Putative Physiopathological Roles. Int. J. Mol. Sci..

[13] Briones-Orta M.A., Avendaño-Vázquez S.E., Aparicio-Bautista D.I., Coombes J.D., Weber G.F., Syn W.-K. (2017). Osteopontin Splice Variants and Polymorphisms in Cancer Progression and Prognosis. Biochim. Biophys. Acta Rev. Cancer.

[14] Silva G.R., Mattos D.S., Bastos A.C.F., Viana B.P.P.B., Brum M.C.M., Ferreira L.B., Gimba E.R.P. (2020). Osteopontin-4 and Osteopontin-5 Splice Variants Are Expressed in Several Tumor Cell Lines. Mol. Biol. Rep..

[15] Briese J., Cheng S., Ezzat S., Liu W., Winer D., Wagener C., Bamberger A.-M., Asa S. (2010). Osteopontin (OPN) Expression in Thyroid Carcinoma. Anticancer Res..

[16] Meireles A.M., Preto A., Rocha A.S., Rebocho A.P., Máximo V., Pereira-Castro I., Moreira S., Feijão T., Botelho T., Marques R. (2007). Molecular and Genotypic Characterization of Human Thyroid Follicular Cell Carcinoma-Derived Cell Lines. Thyroid.

[17] Ferreira L.B., Tavares C., Pestana A., Pereira C.L., Eloy C., Pinto M.T., Castro P., Batista R., Rios E., Sobrinho-Simões M. (2016). Osteopontin-a Splice Variant Is Overexpressed in Papillary Thyroid Carcinoma and Modulates Invasive Behavior. Oncotarget.

[18] Hosni I., Iles A., Greenman J., Wade M.A. (2023). A Robust, Flow-Based, Microfluidic Device for siRNA-Mediated Gene Knockdown in Glioblastoma Spheroids. Innov. Emerg. Technol..

[19] Barry A., Samuel S.F., Hosni I., Moursi A., Feugere L., Sennett C.J., Deepak S., Achawal S., Rajaraman C., Iles A. (2023). Investigating the Effects of Arginine Methylation Inhibitors on Microdissected Brain Tumour Biopsies Maintained in a Miniaturised Perfusion System. Lab Chip.

[20] Fierro-Maya L.F., González G.G., Rojas Melo L.J., Cuéllar Cuéllar A.A., Carreño A., Córdoba C. (2021). Safety and Efficacy of Sorafenib in Patients with Advanced Thyroid Carcinoma: A Phase II Study (NCT02084732). Arch. Endocrinol. Metab..

[21] Brose M.S., Nutting C.M., Jarzab B., Elisei R., Siena S., Bastholt L., de la Fouchardiere C., Pacini F., Paschke R., Shong Y.K. (2014). Sorafenib in Radioactive Iodine-Refractory, Locally Advanced or Metastatic Differentiated Thyroid Cancer: A Randomised, Double-Blind, Phase 3 Trial. Lancet.

[22] Mammatas L.H., Zandvliet A.S., Rovithi M., Honeywell R.J., Swart E.L., Peters G.J., Menke-van der Houven van Oordt C.W., Verheul H.M.W. (2020). Sorafenib Administered Using a High-Dose, Pulsatile Regimen in Patients with Advanced Solid Malignancies: A Phase I Exposure Escalation Study. Cancer Chemother. Pharmacol..

[23] Hao C., Cui Y., Owen S., Li W., Cheng S., Jiang W.G. (2017). Human Osteopontin: Potential Clinical Applications in Cancer (Review). Int. J. Mol. Med..

[24] Lin J., Myers A.L., Wang Z., Nancarrow D.J., Ferrer-Torres D., Handlogten A., Leverenz K., Bao J., Thomas D.G., Wang T.D. (2015). Osteopontin (OPN/SPP1) Isoforms Collectively Enhance Tumor Cell Invasion and Dissemination in Esophageal Adenocarcinoma. Oncotarget.

[25] Nitschke L., Hu R.-C., Miller A.N., Lucas L., Cooper T.A. (2023). Alternative Splicing Mediates the Compensatory Upregulation of MBNL2 upon MBNL1 Loss-of-Function. Nucleic Acids Res..

[26] Kurata M., Yamazaki Y., Kanno Y., Ishibashi S., Takahara T., Kitagawa M., Nakamura T. (2011). Anti-Apoptotic Function of Xbp1 as an IL-3 Signaling Molecule in Hematopoietic Cells. Cell Death Dis..

[27] Ho N.-T., Lin S.-W., Lee Y.-R., Tzeng C.-R., Kao S.-H. (2022). Osteopontin Splicing Isoforms Contribute to Endometriotic Proliferation, Migration, and Epithelial-Mesenchymal Transition in Endometrial Epithelial Cells. Int. J. Mol. Sci..

[28] Anborgh P.H., Mutrie J.C., Tuck A.B., Chambers A.F. (2011). Pre-and Post-Translational Regulation of Osteopontin in Cancer. J. Cell Commun. Signal..

[29] Collins A.L., Rock J., Malhotra L., Frankel W.L., Bloomston M. (2012). Osteopontin Expression Is Associated with Improved Survival in Patients with Pancreatic Adenocarcinoma. Ann. Surg. Oncol..

[30] Ferreira L.B., Eloy C., Pestana A., Lyra J., Moura M., Prazeres H., Tavares C., Sobrinho-Simões M., Gimba E., Soares P. (2016). Osteopontin Expression Is Correlated with Differentiation and Good Prognosis in Medullary Thyroid Carcinoma. Eur. J. Endocrinol..

[31] Lee J.M., Mhawech-Fauceglia P., Lee N., Parsanian L.C., Lin Y.G., Gayther S.A., Lawrenson K. (2013). A Three-Dimensional Microenvironment Alters Protein Expression and Chemosensitivity of Epithelial Ovarian Cancer Cells in Vitro. Lab. Investig..

[32] Ghosh S., Spagnoli G.C., Martin I., Ploegert S., Demougin P., Heberer M., Reschner A. (2005). Three-Dimensional Culture of Melanoma Cells Profoundly Affects Gene Expression Profile: A High Density Oligonucleotide Array Study. J. Cell. Physiol..

[33] Kenny P.A., Lee G.Y., Myers C.A., Neve R.M., Semeiks J.R., Spellman P.T., Lorenz K., Lee E.H., Barcellos-Hoff M.H., Petersen O.W. (2007). The Morphologies of Breast Cancer Cell Lines in Three-Dimensional Assays Correlate with Their Profiles of Gene Expression. Mol. Oncol..

[34] Lipreri M.V., Totaro M.T., Baldini N., Avnet S. (2025). From Spheroids to Tumor-on-a-Chip for Cancer Modeling and Therapeutic Testing. Micromachines.

[35] Bonnet V., Angelidakis E., Sart S., Baroud C.N. (2025). Microfluidic and Organ-on-a-Chip Approaches to Model the Tumor Microenvironment. Curr. Opin. Biomed. Eng..

[36] Nakagawa R., Minamiguchi S., Kataoka T.R., Fujikura J., Masui T., Fujimoto M., Yamada Y., Takeuchi Y., Teramoto Y., Ito H. (2023). Circularity of Islets Is a Distinct Marker for the Pathological Diagnosis of Adult Non-Neoplastic Hyperinsulinemic Hypoglycemia Using Surgical Specimens. Diagn. Pathol..

[37] Wang L.X., Liu S.L., Wu N. (2025). Application and Development of Organ-on-a-Chip Technology in Cancer Therapy. Front. Oncol..

[38] Picca F., Giannotta C., Tao J., Giordanengo L., Munir H.M.W., Botta V., Merlini A., Mogavero A., Garbo E., Poletto S. (2024). From Cancer to Immune Organoids: Innovative Preclinical Models to Dissect the Crosstalk between Cancer Cells and the Tumor Microenvironment. Int. J. Mol. Sci..

[39] BRAF-AXL-PD-L1 Signaling Axis as a Possible Biological Marker for RAI Treatment in the Thyroid Cancer ATA Intermediate Risk Category. https://www.mdpi.com/1422-0067/24/12/10024.

[40] Kim S.Y., Kim S.-M., Chang H., Chang H.-S., Park C.S., Lee Y.S. (2020). Synergistic Anticancer Activity of Sorafenib, Paclitaxel, and Radiation Therapy on Anaplastic Thyroid Cancer in Vitro and in Vivo. Head Neck.

[41] Zhu W., Xie B. (2023). PLK4 Inhibitor Exhibits Antitumor Effect and Synergizes Sorafenib via Arresting Cell Cycle and Inactivating Wnt/β-Catenin Pathway in Anaplastic Thyroid Cancer. Cancer Biol. Ther..

