# Supplementary material for: Osteopontin-4 (OPN-4) Suppresses Tumor Progression Features Whilst Sensitizing c643 Anaplastic Thyroid Cells to Sorafenib
Source: Biomedicines. 2026 Apr 25;14(5):989. doi: 10.3390/biomedicines14050989 (PMC13203564; doi:10.3390/biomedicines14050989)
Supplement: Supplementary file 1 [file biomedicines-14-00989-s001.zip › biomedicines-4160760-supplementary.pdf]

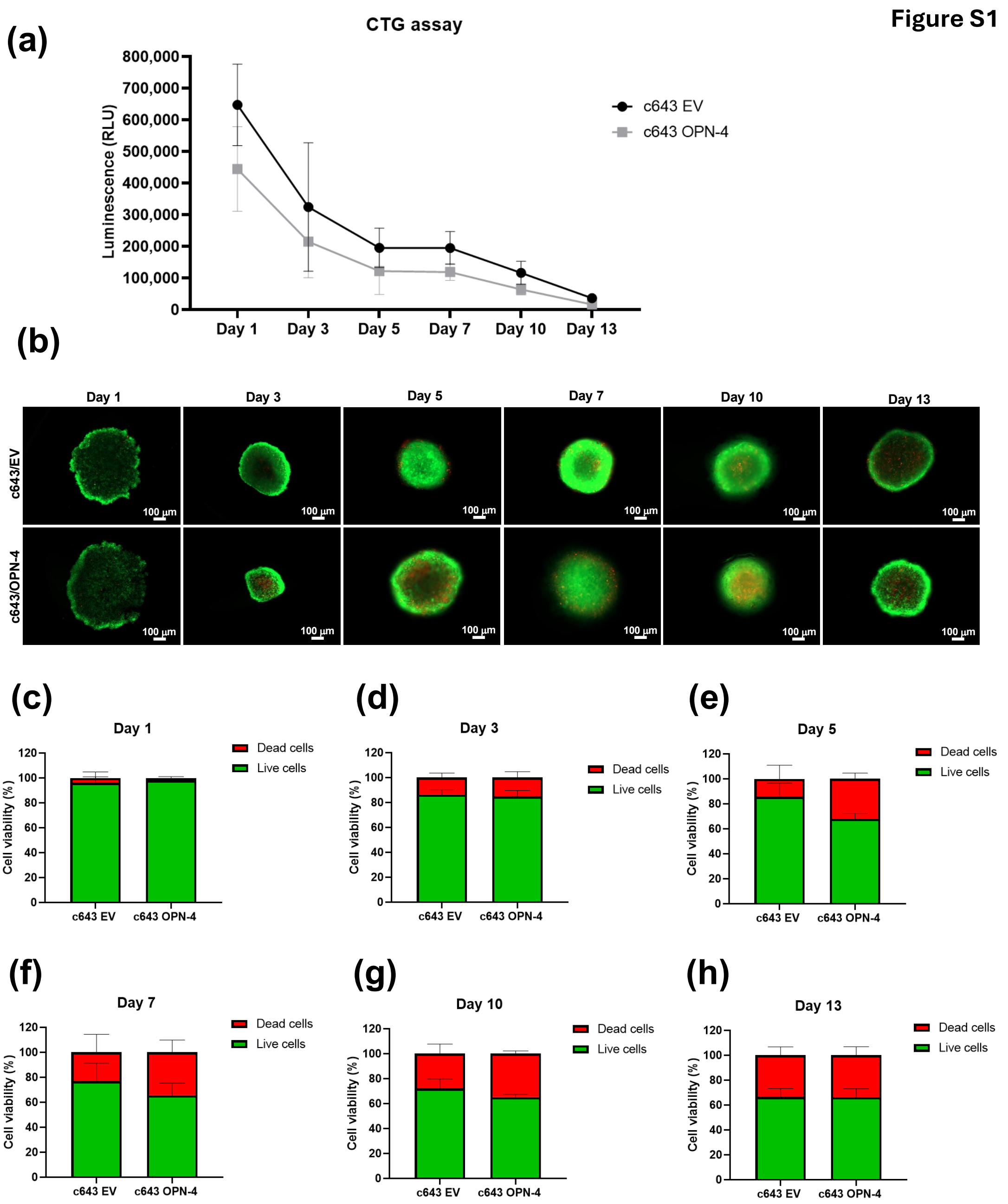

Figure S1. Viability analysis of 3D tumor spheroids derived from OPN-4-overexpressing cells. (a) Luminescence values from the CellTiter-Glo (CTG) assay for c643/EV and c643/OPN-4 spheroids measured over time. (b) Representative fluorescent images of spheroids stained with Fluorescein Diacetate (FDA; live cells, green) and Propidium Iodide (PI; dead cells, red) at multiple time points. (c–h) Quantification of live/dead cell ratios at (c) day 1; (d) day 3; (e) day 5, (f) day 7; (g) day 10, and (h) day 13. ImageJ was used to calculate the percentage of viable versus non-viable cells. Data rep- resents the mean ± SD from three independent experiments. Statistical analysis was performed us- ing an unpaired t-test. Scale bar = 100 µm.

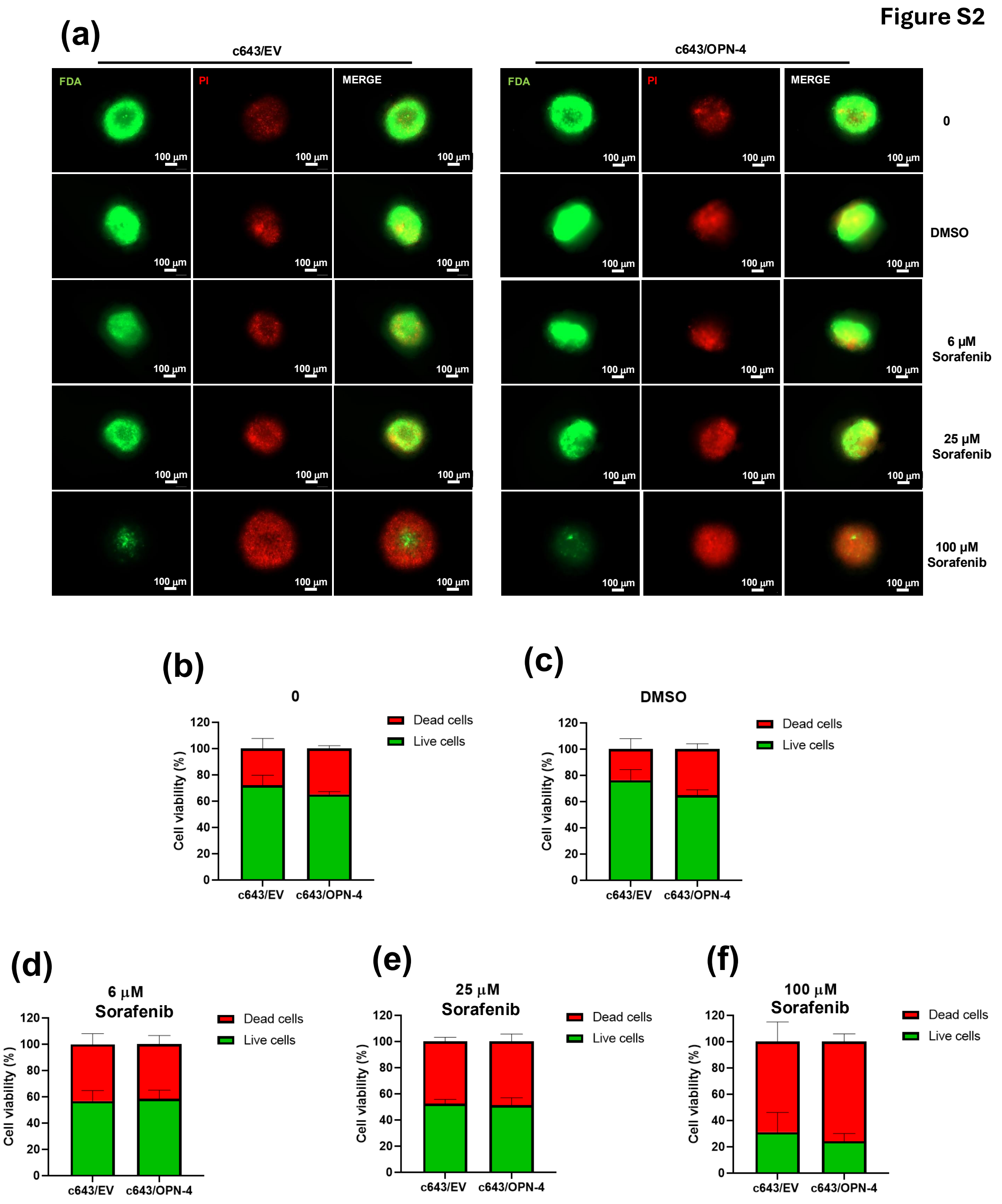

Figure S2. Evaluation of static sorafenib treatment in plate-cultured OPN-4-overexpressing tumor spheroids. (a) Representative fluorescent images of spheroids stained with Fluorescein Diacetate (FDA; live cells, green) and Propidium Iodide (PI; dead cells, red) following 48h of sorafenib treat- ment. Images were acquired using a fluorescence microscope at 10× magnification. Each image rep- resents the result of a representative assay from a total of three independent experiments; (b-f) Quantification of live/dead cell ratios in spheroids treated with (b) only culture medium (0 µM); (c) DMSO (vehicle control); (d) 6 µM; (e) 25 µM, and (f) 100 µM sorafenib. ImageJ was used to calculate the percentage of viable versus non-viable cells. Data represent the mean ± SD from three independ- ent experiments. Statistical analysis was performed using an unpaired t-test. Scale bar = 100 µm.
